# Autocrine IL-6 mediates pituitary tumor senescence

**DOI:** 10.18632/oncotarget.13577

**Published:** 2016-11-24

**Authors:** Melanie Sapochnik, Mariana R Haedo, Mariana Fuertes, Pablo Ajler, Guillermo Carrizo, Andrés Cervio, Gustavo Sevlever, Günter K. Stalla, Eduardo Arzt

**Affiliations:** ^1^ Instituto de Investigación en Biomedicina de Buenos Aires (IBioBA)-CONICET-Partner Institute of the Max Planck Society, Buenos Aires, C1425FQD, Argentina; ^2^ Servicio de Neurocirugía, Hospital Italiano, C1199ABD, Buenos Aires, Argentina; ^3^ Departamento de Neurocirugía, Fundación para la Lucha contra las Enfermedades Neurológicas de la Infancia (FLENI), C1428AQK, Buenos Aires, Argentina; ^4^ Department of Clinical Research, Max Planck Institute of Psychiatry, Munich, Germany; ^5^ Departamento de Fisiología y Biología Molecular y Celular, Facultad de Ciencias Exactas y Naturales, Universidad de Buenos Aires, Buenos Aires, C1428EGA, Argentina

**Keywords:** senescence, IL-6, pituitary tumor, benign tumor, autocrine

## Abstract

Cellular senescence is a stable proliferative arrest state. Pituitary adenomas are frequent and mostly benign, but the mechanism for this remains unknown. IL-6 is involved in pituitary tumor progression and is produced by the tumoral cells. In a cell autonomous fashion, IL-6 participates in oncogene-induced senescence in transduced human melanocytes. Here we prove that autocrine IL-6 participates in pituitary tumor senescence. Endogenous IL-6 inhibition in somatotroph MtT/S shRNA stable clones results in decreased SA-β-gal activity and p16^INK4a^ but increased pRb, proliferation and invasion. Nude mice injected with IL-6 silenced clones develop tumors contrary to MtT/S wild type that do not, demonstrating that clones that escape senescence are capable of becoming tumorigenic. When endogenous IL-6 is silenced, cell cultures derived from positive SA-β-gal human tumor samples decrease the expression of the senescence marker. Our results establish that IL-6 contributes to maintain senescence by its autocrine action, providing a natural model of IL-6 mediated benign adenoma senescence.

## INTRODUCTION

Cellular senescence is a state of stable proliferative arrest in G_1_ phase of the cell cycle through activation of the p53/p21^Cip1^ and pRb/p16^INK4a^ signaling pathways, including alterations in the phenotype of the cells. Senescence is induced by several cellular events such as telomere dysfunction, cellular stress, DNA and chemical damage or oncogene activation [1–3]. Senescence includes several different effector mechanisms and has been recently described not only as a static endpoint, but also as a dynamic process that ends in phenotypic changes [4, 5]. This feature is more relevant in acute types of senescence, such as oncogene-induced senescence (OIS), where the initial proliferative-mitotic state mimics transformation, but is gradually replaced by senescence [6]. Thus, OIS represents the action of senescence as a tumor-suppresive mechanism [7, 8]. OIS has been implicated in the arrest of several types of benign tumors, such as human and murine melanocytic nevi [9, 10], human dermal neurofibromas [11] and human schawnnomas [12], but not in malignant adenocarcinomas.

Pituitary adenomas are usually benign, non-metastatic and monoclonal tumors formed by adeno-pituitary gland cells, which cause small local lesions and display a slow growth rate [13–15]. The mechanisms for the particular benign feature of these adenomas are still not clear. Since pituitary adenomas have exhibited stable growth after decades of observation [16] and senescence occurs in early stages of benign but not in malignant tumors, it could be proposed as a major mechanism to understand its benign nature [17]. Pituitary adenoma senescent phenotype [18, 19] and the involvement of the oncogene *pttg* [18], have been observed. Thus, initial pituitary tumor senescence may circumvent pro-proliferative signals, contributing to stop cell proliferation, but preserving important homeostatic pituitary functions [14, 17, 20].

Normal pituitary cells are under the auto-/paracrine control of several growth factors. Changes in the expression levels and/or the function of these molecules have been described to contribute to pituitary adenoma development [21]. Altered levels of cytokines and growth factors and their corresponding receptors, such as transforming growth factor alfa and beta protein families, epidermal growth factor, fibroblast growth factor family, bone morphogenetic protein 4 and interleukin 6 (IL-6)/glycoprotein 130 family, has been observed in pituitary tumors [21–24]. In particular, it is known that IL-6 participates in pituitary tumor development and progression. It is produced by the tumoral cells but is also secreted to the normal or adenoma cells by folliculo stellate (FS) cells, which mix up with the normal pituitary cells and further surround the pituitary tumors [25–28]. In pituitary adenoma cultures, cells other than FS cells are responsible for IL-6 production [29]. It has been shown that IL-6 inhibits normal pituitary cell proliferation and has opposite effects in normal and tumoral pituitary cells [30, 31].

A previous report [32] showed that IL-6 plays an important role in OIS induction and maintenance in transduced human melanocytes, acting in a cell-autonomous mode to allow OIS. Autocrine IL-6 acts to regulate OIS and the same senescent cells produce an IL-6 pool that acts promitogenically in a paracrine way, indicating that IL-6 can function as an autocrine or paracrine tumorigenic factor. Taking into account that IL-6 participates in the progression of pituitary tumors, and its role in OIS, this cytokine appears as a candidate for an autocrine/paracrine regulator of pituitary adenoma control [17, 30, 33].

In the present work we demonstrate that IL-6 participates in pituitary tumoral senescence. Our findings establish that IL-6 contributes to maintain senescence by its autocrine action, providing a natural model of IL-6 mediated tumor senescence, which may contribute to explain the benign behavior of these abundant adenomas.

## RESULTS AND DISCUSSION

### Assessment of senescence biomarkers in pituitary tumor cell lines and characterization of MtT/S cells senescent phenotype

**Figure 1:**
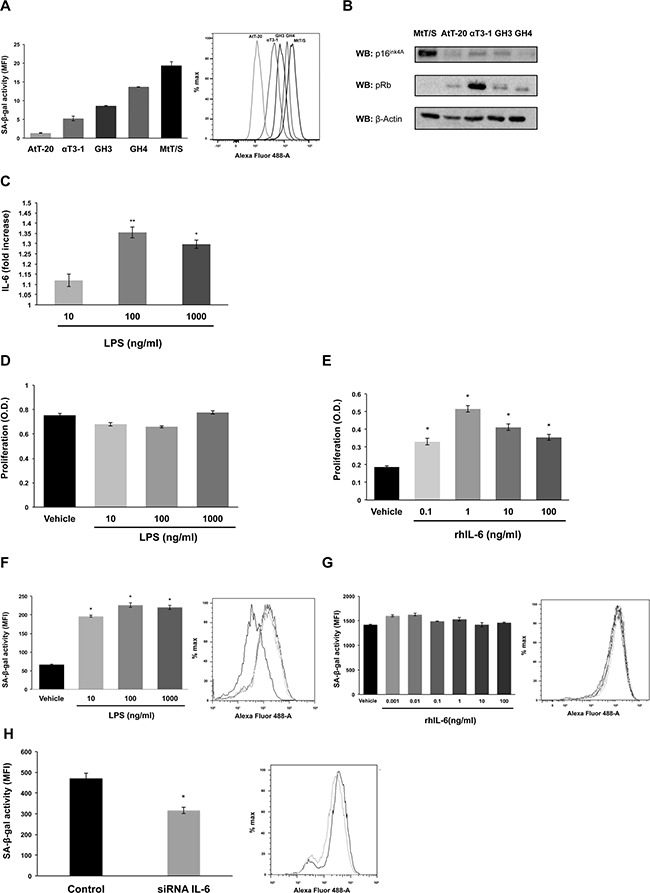
MtT/S pituitary cell line presents a senescent phenotype modulated by endogenous IL-6 **A**. SA-β-gal activity was measured by flow cytometry. The bar graph represents the SA-β-gal activity measured by the mean fluorescence intensity (MFI). **B**. To assay pRb and p16^INK4a^ protein levels, cell extracts were subjected to western blot (WB) with the indicated antibodies. β-Actin was used as loading control. **C**. IL-6 secretion by MtT/S cells under basal and stimulated conditions with LPS was measured by ELISA. Values represent the average fold stimulation with respect to basal. Absolute values of basal (similar to vehicle) IL-6: 72.71±0.74 pg/ml (30,000 cells/well). **D-E**. Effect of LPS and rhIL-6 on MtT/S cell proliferation, measured with the WST-1 assay (A_450nm_). **F-H**. Effect of LPS, rhIL-6 and siRNA IL-6 on MtT/S SA-β-gal activity. The grey scale used in the histogram plot correspond to the grey scale used in the bar graph, which represent the SA-β-gal activity measured by the mean fluorescence intensity (MFI). One representative experiment from three independent experiments (N= 4 for each condition in each experiment) with similar results is shown. Results are expressed as mean ± SEM. *p<0.05 and **p<0.01 compared with cells treated with vehicle (C-G) or cells transfected with siRNA GL3 as control (H). ANOVA with Scheffé's test.

We first explored whether a pituitary tumor cell line could serve as a suitable model for studies of senescence in pituitary tumors. For this, we analyzed the senescent markers, senescence-associated beta-galactosidase (SA-β-gal) and pRb/p16^INK4a^ in several pituitary tumor cell lines (MtT/S, αT3-1, AtT-20, GH3 and GH4), and found that MtT/S cells present a clear senescent phenotype. These cells have increased SA-β-gal activity (Figure 1A) and p16^INK4a^ expression (Figure 1B) and, consequently, decreased pRb expression (Figure 1B). Previous research performed on the MtT/S cell line showed that it cannot develop tumors unless exogenous IL-6 is present [34]. In contrast, it was also shown that the injection of the other cell lines, that do not have a senescent phenotype, i.e. AtT-20 [35], αT3-1 [36], GH3 [22] and GH4 [37], develop tumors in nude mice. These findings indicate that the somatotrophic MtT/S cell line presents senescent features. The study of senescence *in vitro* makes use of cell lines which reached, or are near to reach [38, 39], their Hayflick limit or that were manipulated to develop the phenotype [32, 38, 40]. It has been observed that the mouse pituitary gonadotroph cell line LβT2 presents some senescent features which were increased by the overexpression of PTTG [41]. Our findings show that the rat somatotroph cell line MtT/S presents a senescent phenotype, and constitutes an interesting model of pituitary tumor senescence.

Based on the described role of IL-6 in OIS [32], we analyzed whether these cells produce IL-6 and its role in growth versus senescent phenotype. MtT/S cells increase IL-6 production 10-35% after LPS stimulation (Figure 1C). LPS treatment with concentrations that increased IL-6 production did not affect MtT/S cell proliferation (Figure 1D), showing that endogenous production of IL-6 does not affect proliferation. On the contrary, stimulation with exogenous IL-6 increased MtT/S cell proliferation (Figure 1E). IL-6 exerts the maximum effect at 1 ng/ml, higher doses do not have a greater effect, but still induce proliferation, probably because of saturating the receptors. Incubation of MtT/S cells with LPS concentrations that increased their IL-6 production, and do not affect its proliferation, led to an increase in SA-β-gal activity (Figure 1F). Incubation with exogenous IL-6, on the other hand, did not affect SA-β-gal activity (Figure 1G). Interestingly, even adding IL-6 at similar concentrations to those produced by MtT/S under LPS stimulation have not effect on SA-β-gal, showing that the source of IL-6 is critical for its action.

In order to verify that the endogenous IL-6 directly regulates SA-β-gal activity, we silenced IL-6 in MtT/S cells with a siRNA directed specifically towards rat IL-6 (Supplementary Figure 1A). Transfection with this siRNA resulted in decreased in SA-β-gal activity (Figure 1H).

### Generation of MtT/S shIL-6 clones and role of IL-6 in pituitary MtT/S senescence *in vivo*

To further characterize the role of IL-6, we generated clones of MtT/S by stable transfection with plasmids coding simultaneously for shRNA targeting rat IL-6 and eGFP. The insertion of the plasmid and the IL-6 silencing was verified by confocal microscopy and by measuring mRNA levels and protein secretion (Supplementary Figures 1B, 1C and 1D), and STAT3 phosphorylation was measured to confirm that IL-6 silencing was sufficient to affect its biological function (Supplementary Figure 1E). GH expression shows a reduction in MtT/S shIL-6 clones (Supplementary Figure 1F), which is in accordance with the known stimulatory action of IL-6 on GH [30, 33] and the recently described stimulation of STAT3 on GH [42]. All the experiments were performed in three independent clones (for both shRNA IL-6 and unspecific shRNA) and similar results were obtained.

**Figure 2:**
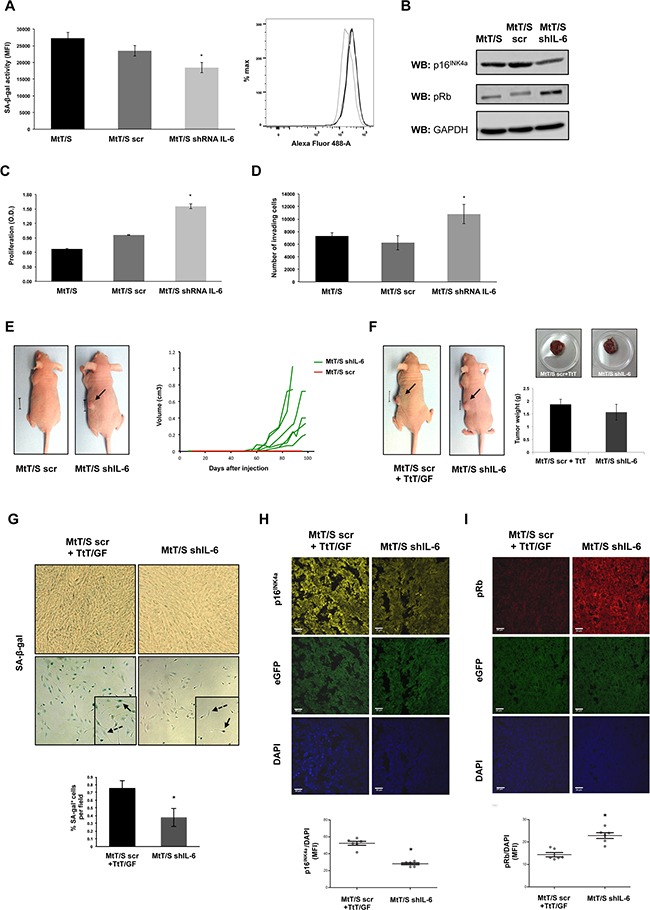
IL-6 silenced clones present decreased senescent biomarkers and acquire the capacity to form tumors **A**. SA-β-gal activity was measured on MtT/S wild type (MtT/S), MtT/S control (MtT/S scr) and MtT/S IL-6 silenced (MtT/S shIL-6) clones, by flow cytometry. The bar graph represents the SA-β-gal activity measured by the mean fluorescence intensity (MFI). **B**. For pRb and p16^INK4a^ protein levels, cell extracts were subjected to western blot (WB) with the indicated antibodies. GAPDH was used as loading control. **C-D**. Cell proliferation, measured by WST-1 assay (A_450nm_), and cell invasion, measured using transwells, were performed on MtT/S, MtT/S scr and MtT/S shIL-6 clones. One representative experiment from three independent experiments (N= 3 for each condition in each experiment) with similar results is shown (A-D). Results are expressed as mean ± SEM. *p<0.05 compared with MtT/S and MtT/S scr, ANOVA with Scheffé's test (A, C and D). **E**. MtT/S scr and MtT/S shIL-6 clones were injected subcutaneously (5×10^5^ cells for each clone). Photographs were taken at 70 days. One mouse with similar results to all the others of each group is shown (i.e. no tumor in all the animals injected with MtT/S scr cells and tumors similar to the one shown in the picture in all animals injected with MtT/S shIL-6). Volume of each of the tumors of one of three independent experiments with six mice in each group in each experiment with similar results is shown. The six MtT/S scr mice did not form tumors and overlap in one red line and the volume from each of the tumors from the MtT/S shIL-6 group is shown in green. **F**. MtT/S scr with TtT/GF and MtT/S shIL-6 cells were injected subcutaneously in 2 groups of five male nude mice (5×10^5^ cells for each clone and 1,8×10^5^ cell for TtT/GF). Photographs of mice were taken at 70 days. Tumor weights were obtained at the end of the experiment. One mouse and one tumor with similar results to the others of each group are shown. Three independent experiments with five mice in each group in each experiment were performed, showing similar results. Weights of one of these experiments are shown in the graph. **G**. 6μm tumor cryosections (upper panel, x20 magnification) and primary cell cultures derived from the same tumor (lower panel, x10 magnification) were stained for SA-β-gal. Arrows and dotted line arrows indicate examples of SA-β-gal positive and negative cells, respectively. Quantification of positive SA-β-gal cells is shown. Results obtained from 20 independent pictures for each condition are expressed as mean ± SEM. **H-I**. 6μm tumor sections were stained by double immunofluorescence with p16^INK4a^ (yellow; 1:50), pRb (red; 1:50) and 4′,6-diamidino-2-phenylindole (DAPI) for staining cell nuclei (blue). Tumors developed by the injected cells were verified by the detection of eGFP (green). Images were acquired at x63 magnification (scale bar: 20μm). p16^INK4a^ and pRb were quantified by the mean fluorescence intensity (MFI) and relativized to DAPI signal. Results obtained from six independent pictures for each condition are expressed as mean ± SEM. One representative picture for each condition is shown. *p<0.05 compared with tumors developed by the co-injection of MtT/S scr and TtT/GF, Student t test (H-I).

In these clones we first analyzed SA-β-gal and, in line with our previous results, we observed that IL-6 silenced clones present decreased SA-β-gal activity (Figure 2A). Further we studied the expression of the cell cycle regulators p16^INK4a^ and pRb, observing decreased p16^INK4a^ but increased expression of pRb compared to wild type and control-scramble plasmid transfected cells (MtT/S scr) (Figure 2B). Stable silencing of IL-6 produced a significant increase in proliferation (Figure 2C) and invasive capacity of MtT/S cells (Figure 2D). There are different IL-6 signaling pathways described [43, 44] that can account for the autocrine and paracrine differential signaling all of which involve STAT3 activation, but also integrate different cross-talk pathways that may explain the differential action on proliferation and senescence. An important role for gp130/STAT3 has been shown in corticotroph cells [45, 46], and might be involved on the IL-6 action observed in these cells.

In order to prove that endogenous IL-6 induces senescence and avoids the development of MtT/S cells into tumors, we injected the MtT/S shIL-6 clones into nude mice. As described for the MtT/S wild type cells [34], MtT/S scr did not form tumors (Figure 2E). However, mice injected with the IL-6 silenced clones developed tumors that started to be observable at 35-45 days after being injected (Figure 2E). This result indicates that these clones, that escape senescence *in vitro*, are able to enter tumorigenesis *in vivo*.

MtT/S wild type cells co-injected with TtT/GF cells, due to the production of IL-6 by these cells, are able to develop tumors [34, 47]. In order to analyze the impact of the IL-6 silencing on the senescence biomarkers *in vivo*, and taking into account that MtT/S wild type or scramble do not establish tumors, we compared the senescent markers of the tumors developed by the co-injection of MtT/S scr with IL-6 producing TtT/GF cells and those produced by the cells in which endogenous IL-6 is silenced, that show similar sizes and weights (Figure 2F). We observed that tumors developed by MtT/S shIL-6 clones present a lower SA-b-gal activity (Figure 2G), lower expression of p16^INK4a^ (Figure 2H) and higher expression of pRb (Figure 2I) than the ones generated by the co-injection of MtT/S scr and TtT/GF.

The facts that MtT/S cells do not generate tumors and that the absence of endogenous IL-6 allowed them to bypass senescence and consequently develop into tumors, support the idea that IL-6 by its autocrine action contribute to maintain the senescent phenotype of these tumoral cells. To determine IL-6 autocrine action *in vivo*, we compared senescent markers in tumors expressing (MtT/S scr cells co-injected with TtT/GF cells) or lacking (MtT/S shIL-6) endogenous IL-6. Thus we were able to compare the tumor developed by the silencing of IL-6 (i.e. the abolishment of senescence) with a tumor resembling the natural situation in which both the paracrine proliferative IL-6 and the autocrine-inducing senescence are on. In support of the notion about the role of IL-6, the tumors expressing endogenous IL-6 present a more pronounced senescent phenotype.

### Role of IL-6 in senescence of human pituitary adenomas

**Figure 3:**
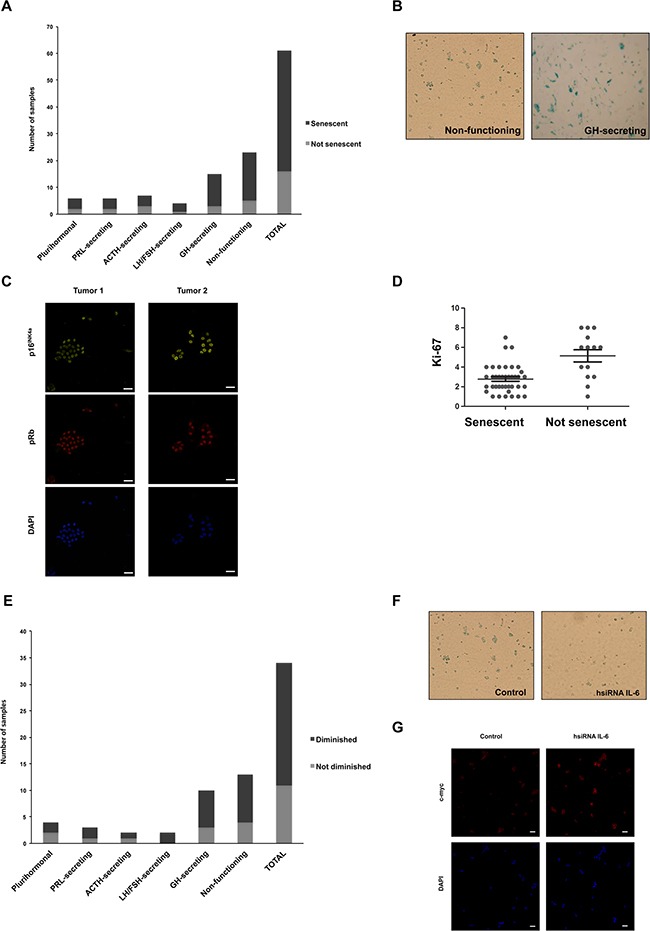
Human pituitary tumor samples express SA-b-gal modulated by IL-6 **A**. Primary human pituitary cell cultures prepared from 61 samples of human pituitary tumor samples were stained for SA-β-gal (6 plurihormonal, 6 PRL-secreting, 7 ACTH-secreting, 4 LH/FSH-secreting, 15 GH-secreting and 23 non-functioning tumors). Black zone of the bars represents positive samples, while grey zone represents negative samples. **B**. Photographs of positive SA-β-gal staining from a non-functioning and a GH-secreting tumor are shown. **C**. Photographs of positive p16^INK4a^ (yellow; 1:50) and pRb (red, 1:50) staining from a non-functioning and a GH-secreting tumor are shown. 4′,6-diamidino-2-phenylindole (DAPI) were used for staining cell nuclei (blue). Images were acquired at x63 magnification (scale bar: 20μm). **D**. By Pearson correlation analysis it was determined that senescence correlates negatively with the cellular marker of proliferation Ki-67 in human pituitary adenomas (Pearson coefficient=-0.3245, p<0.05). **E**. Positive samples of human pituitary tumors (N=34) were electroporeted with 10mM hsiRNA IL-6 or 10mM siRNA GL3 and then stained for SA-β-gal. Black zone of the bars represents samples in which SA-β-gal staining were significantly diminished (Student t test, p<0.05), while grey zone represents samples in which SA-β-gal staining did not vary. **F**. Photographs of a representative non-functioning tumor with significant decrease in SA-β-gal with hsiRNA IL-6 are shown. Images were acquired at x40 magnification. **G**. Photographs of a representative non-functioning tumor with significant increase in c-myc (red, 1:50) with hsiRNA IL-6 are shown. 4′,6-diamidino-2-phenylindole (DAPI) were used for staining cell nuclei (blue). Images were acquired at x40 magnification (scale bar: 20μm).

To characterize the senescent phenotype of the human pituitary adenomas, and confirming previous reports of senescence in pituitary tumors [18, 19], SA-β-gal staining was performed on primary cell cultures prepared from 61 adenoma tissue samples. 74% (45/61) of the analyzed samples were positive for the SA-β-gal staining (Figure 3A and 3B). In 6 of these positive samples we further analyzed p16^INK4a^ and pRb and they all express these other senescence markers (Figure 3C). We found a negative correlation between senescence and the cellular marker of proliferation Ki-67 (Figure 3D), suggesting that, as hypothesized, senescence prevents proliferation of pituitary tumors. The higher incidence of senescence was found in GH-secreting (12/15) and non-functioning (NF) tumors (18/23), as previously observed [19]. Of note, none of the GH senescent adenomas and only 10% of the total senescent tumors were invasive.

**Figure 4:**
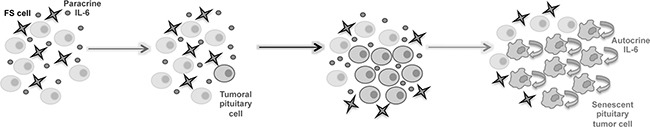
Pituitary senescence progression model Pituitary tumor pathogenesis has an initial proliferative phase involving IL-6 secreted from the FS cells, which induces proliferation of the pituitary tumor cells. It is followed by a phase of stopping proliferation in which senescence mediated by the autocrine IL-6 produced by the tumor cells themselves, might contribute to establish a benign tumor with stable growth arrest.

Primary cell cultures derived from positive SA-β-gal staining tumor samples (Figure 3A) were electroporated with siRNA targeting human IL-6 (hsiRNA IL-6) or unspecific siRNA as control. The levels of IL-6 in the tumors after silencing were verified with a fluorescent-coupled oligonucleotide and by immunofluorescence with a specific antibody against IL-6 (Supplementary Figures 2A and 2B). As shown in Figure 3E and 3F, 68% (23/34) presented a significant reduction in the SA-β-gal staining when endogenous IL-6 was decreased. The IL-6 siRNA also produced an increase in the expression of c-myc (Figure 3G).

We describe here that IL-6, beside its previously known action on proliferation and regulation of GH (as observed in the MtT/S shIL-6 clones), plays a fundamental role in senescence in pituitary tumors, contributing to explain their benign characteristics. Regardless of the abundance of pituitary adenomas [48], they are not commonly metastatic [49–52]. The protective role of OIS may provide an interesting mechanism to further understand this behavior. OIS demands time to develop, enabling an initial proliferative phase, that results in a benign tumor with stable growth arrest, which is the natural history of most of pituitary adenomas. Initial studies described senescence and PTTG involvement in human pituitary adenomas [18, 19], although the mechanisms underlying this behavior remain an open question. Different to most human GH-producing pituitary adenomas in which PTTG overexpression is associated with p21-dependent senescence [19], tumors arising from the gonadotroph lineage also exhibit high PTTG levels, but p21 is not expressed in gonadotroph-derived non-functioning pituitary adenomas, which express p15^INK4b^ and p16^INK4a^, pointing to a differential lineage-specific pathways restricting and controlling pituitary cell cycle progression [41]. Epigenetic changes, such as demethylation of specific genes [53], that may also occur on cytokines [54], or cell cycle regulators [55, 56], might also be involved in the cascade of events leading to the senescence phenotype of pituitary tumors. Given its role both in pituitary pathophysiology [24, 30, 33, 57, 58] and in OIS [32], we focused on IL-6 as a potential candidate for mediating this processes during pituitary adenoma development.

The pituitary has a considerable degree of plasticity and self-renewal capacity. Recent progress described the characterization of adult pituitary stem cells (SCs) [59–62]. Several cell types and populations, including FS cells, have been considered to represent SCs [63–65]. Current data suggests that SCs have a tumorigenic potential. The initial mutation that drives pituitary tumorigenesis may occur in SCs and through the secretion of mitogenic signals (interleukins, chemokines and growth factors) induce the transformation of surrounding cells that will form a tumor [66]. Paracrine IL-6 is one of the factors that these cell types, which do not form part of the tumor, may secrete.

In the normal pituitary paracrine IL-6 delivered by FS cells may act to induce the development of an adenoma, by promoting tumor cell expansion (Figure 4). In line with this, previous works have proven that the MtT/S cell line depends on the folliculostellate TtT/GF cell line to produce tumors in nude mice [34, 47]. IL-6 secreted by the FS that surround the tumoral cells may also induce extracellular matrix remodeling and vessel formation [25]. After mutation of a normal pituitary cell, autocrine IL-6 in the same tumor may trigger senescence and contribute in this way to control aggressive growth and malignant development of these cells (Figure 4). Several signaling mechanisms for IL-6 have been described, involving a soluble form of the receptor and an endocytosis mechanism [43, 44]. Endogenous IL-6 may involve one of those, or another still unknown pathway, that is not activated by the membrane receptor, e.g. exogenous IL-6. Future research will clarify the interesting open question about the mechanisms involved in the differential action.

Senescence in the pituitary constitutes a mechanism that may contribute not only to restrain proliferation but to allow the pituitary cells to remain viable and accomplish its physiological function, which supports the vital functioning of the pituitary gland for its involvement in the homeostasis control.

Evidence supporting OIS as a physiologically relevant mechanism stopping tumor formation as well as linking to the induction of an inflammatory transcriptome, is rapidly emerging. Our findings demonstrate for the first time the involvement of IL-6 in a natural tumor model.

## MATERIALS AND METHODS

### Materials

Unless otherwise stated, reagents were obtained from Life Technologies or Sigma Chemical Co.

### Cell culture and stimulation

Cell lines AtT-20, GH3 and GH4 acquired from ATCC, either directly or by colleagues, MtT/S and TtT/GF directly obtained from K. Inoue who generated the cells as described below [67, 68], and aT3-1 provided by P. Mellon who generated them [69], were kept frozen immediately after receipt and used each cycle in culture for less than 4 months. ATCC cell lines are characterized by Short Tandem Repeat (STR) profiling. MtT/S cells, a rat somatotrophic pituitary cell line obtained from an estrogen-induced somatotrophic tumor [67], and TtT/GF, a murine folliculostellate cell line obtained from a thyrotrophic pituitary tumor [68], were cultured as described [34]. MtT/S clones, generated as indicated below, were cultured under the same conditions as MtT/S cells but adding Puromycin to the medium. AtT-20, a mouse corticotroph cell line, GH3 and GH4, rat lactosomatotroph cell lines, and αT3-1, a mouse pituitary gonadotroph cell line, were culture as described [23, 69].

Primary human pituitary adenomas and MtT/S shIL-6 or MtT/S scr and TtT/GF tumor cell cultures were performed as described [31, 34]. Briefly, the tissue was washed several times with preparation buffer (137 mmol/L NaCI, 5 mmol/L KCl, 0.7 mol/L Na_2_HPO_4_, 10 mmol/L glucose, 15 mmol/L HEPES, pH 7.3, 2.5 mg/L amphotericin B, and 10^5^ U/L penicillin/streptomycin). Sliced fragments were mechanically and enzymatically dispersed in preparation buffer containing 1000 U/mL collagenase, 4 g/L BSA, 10 mg/L DNAse II, 1 g/L soybean trypsin inhibitor, and 2 g/L hyaluronidase. Cells were centrifuged and resuspended in Dulbecco's modified Eagle's medium (pH 7.3) containing 10% fetal bovine serum, 2.2 g/L NaHCO_3_, 10 mmol/L HEPES, 2 mmol/L glutamine, 10 mL/L nonessential amino acids, 10 mL/L minimal essential medium vitamins, 2.5 mg/L amphotericin B, and 10^5^ U/L penicillin/streptomycin, 5 mg/L insulin, 20 mg/L sodium selenite and 30 pmol/L T_3_. Isolated 2×10^5^ stromal adenoma cells, in absence of FS cells as previously described [70], with a viability of at least 80% (acridine orange/ethidium bromide staining) were cultured under a 5% CO_2_ atmosphere at 37 C.

For LPS and rhIL-6 (R&D Systems, Minneapolis, Minnesota, USA) stimuli, cells were cultured in 2% FBS DMEM for 16 h before stimulation. Cells were treated with indicated doses of LPS or rhIL-6 for 24 h or 72 h, according to the experiment performed. The same diluent (vehicle) was used as control in all experiments.

### Transfections

MtT/S cells transfection with siRNA against rat IL-6 (5′-GAUGGUUUCUUGCAAUAUATT-3′) or unspecific siRNA (5′-CUUACGCUGAGUACUUCGATT-3′) were performed using Lipofectamine 2000 reagent following the manufacturer's instructions.

Stable clones were obtained as described [22]. MtT/S cells were transfected with each shRNA plasmids directed against rat IL-6 from (Kit Hush® shRNA, OriGene, Rockville, Maryland, USA) or an unspecific shRNA (5′-GCACTACCAGAGCTAACTCAGATAGTACT-3′). Plasmids also encode for the green fluorescent protein (GFP). For each shRNA, depending on their proliferative capacity and suitable morphology, 5 clones were selected and amplified. Clones were selected and maintained with 1 μg/ml Puromycin (Invivogen, San Diego, California, USA). Endogenous IL-6 expression levels were checked by RT-PCR, ELISA, and also by checking the expression of eGFP using confocal microscopy. Initial studies showed that all clones had similar results. All experiments were performed in three clones, and results from clone 36 are shown.

Primary human pituitary adenoma cultures were transfected [71], with 100mM siRNA against human IL-6 (5′-CCCAGGAGAAGAUUCCAAATT-3′) or unspecific siRNA, or with BLOCK-it. After electroporation (using a GenePulser from Bio-Rad, Hercules, California, USA) cells were platted, 72 h after, SA-β-staining was performed.

### Reverse transcription–PCR

RT–PCR was performed as previously described [22]. PCR was performed with the specific primers: GAPDH upper primer: 5′-AAGGCTGTGGGCAAGGTCATC-3, GAPDH lower primer: 5′-CGAAGGTGGAAGAGTGGGAGTTG-3′; β-actin upper primer: 5′-GTGGGCCGCTCTAGGCACCA-3′; β-actin lower primer: 5′-CGGTTGGCCTTAGGGTTCAGGGGGG-3′; rat IL-6 upper primer: 5′-ATCTGCTCTGGTCTT CTGG-3′, rat IL-6 lower primer: 5′-GATGAGTTGGATGGT CTTG-3.

### IL-6 secretion

MtT/S and MtT/S clone cells were plated at 80 % confluence. 24 h later medium were collected and stocked at -80 C until IL-6 determination, as described [70]. A rat IL-6 ELISA Kit (R&D Systems) was used.

### Western blot

Cell lysates were prepared in standard cracking buffer and boiled for 5 min at 95 C. Cell extracts were subjected to SDS-polyacrilamyde gel electrophoresis (SDS-PAGE) as previously described [22]. Proteins were blotted onto nitrocellulose membranes using standard procedures, and incubated with p16^INK4a^ (1:1000), STAT3 (1:1000), phosphoSTAT3 (1:1000), β-Actin (1:3000) (Santa Cruz Biotechnologies, California, USA), pRb (1:1000, Cell Signalling Technology, Danvers, Massachusetts, USA), GH (1:1000) or GAPDH (1:10000) (Abcam, Cambridge, Massachusetts, USA) antibodies overnight, followed by corresponding secondary antibodies incubation.

### Cell proliferation

1×10^3^ cells were platted and 72 h later a WST-1 assay Roche Molecular Biochemicals, Mannheim, Germany) was used to measure viability and proliferation following the manufacturer's instructions. The reactions product was measured in an ELISA plate reader at 450 nm, as previously described [23].

### Cell invasion

Invasion assay was performed in 6.5μm Transwells (Corning, New York, USA), that were first coated with Matrigel Matrix (Corning). Cells (5×10^4^) suspended in 100 ml serum-free medium were added to the upper chamber and the lower chamber was filled with DMEM medium with 10% FBS. Cells were allowed to invade at 37 C for 24 h. After removing non-invading cells, membranes were fixed in methanol and stained with 4′,6-diamidino-2-phenylindole (DAPI). Images of invading cells were captured by a LSM 710 AxioObserver fluorescence microscope (Carl-Zeiss, Jena, Germany), acquired with ZEN 2011 software (Carl-Zeiss) and counted with ImageJ.

### *In vivo* experiments in nude mice

MtT/S derived clones (MtT/S scr and MtT/S shIL-6) and TtT/GF were harvest by trypsinization, washed twice with PBS, resuspended in DMEM, and injected subcutaneously, as described [22, 34], into the flanks of 6- to 8-week-old male nude mice (strain N:NIH (S)-FoxnInu), obtained from Fundación Facultad de Ciencias Veterinarios, National University of La Plata, Argentina. Two independent groups of six mice were injected with 5×10^5^ cells of MtT/S scr or MtT/S shIL-6, and two independent groups of five mice were injected with 5×10^5^ cells for each clone and 1.8×10^5^ cell for TtT/GF. Animals were examined for tumor formation every 3 days and tumor growth was determined as described [22, 34]. All experimental protocols were approved by the Ethical Committee on Animal Care and Use (CICUAL), University of Buenos Aires, Argentina.

Nude mice tumors explants were frozen or embedded into paraffin blocks. To prepare cryosections the Microm HM 550 cryostat (Thermo Scientific, Waltham, Massachusetts, USA) was used and to prepare sections of paraffin-embedded, the RM2235 rotary microtome (Leica, Wetzlar, Germany).

### Tumors

Human pituitary adenoma tissues were obtained from the Neurosurgery service, Hospital Italiano, Argentina and Neurosurgery department, FLENI, Argentina. This study complies with the June 1964 Declaration of Helsinki, has been approved by the hospital ethics committee, and informed written consent was received from each patient whose tumor tissue was used in the study.

A portion of the tumor tissue, after transsphenoidal surgery, was received in sterile medium. 61 pituitary adenomas were diagnosed according to clinical, biochemical, radiological and surgical findings as well as by routine immunohistochemistry and were classified as somatotrophinomas (n=15), corticotrophinomas (n=7), prolactinomas (n=6), tyrotrophinomas (n=4), plurihormonal adenomas (n=6) and non-functioning adenomas (n=23), and cultured as described in the cell culture section. All human tumor samples with clinical and immuno-histochemical confirmed diagnosis made at the Hospitals, that have been sent by the neurosurgeons during the time of this study, were included in the first SA-β-gal analysis of this study, no samples obtained were discarded. All positive SA-β-gal samples were electroporated with IL-6 siRNA as described above and further cultured; 34 of these that were able to establish a viable primary culture, were further analyzed.

### SA-β-gal activity

Hystochemical and fluorescence detection of SA-β-gal enzymatic activity were performed as described [72]. Only senescent cells are detected at pH 6.0.

For hystochemical detection, SA-β-gal activity was monitored as the percent of cells with SA-β-gal staining divided by the total number of cells in 20 independent fields. For fluorescence detection using flow cytometry (BD Becton Dockinson FACS CantoII, Heidelberg, Germany), SA-β-gal activity was measured by the mean fluorescence intensity using C_12_FDG as a substrate that becomes fluorescent after cleavage by the enzyme.

### Immunofluorescence

Six-micrometer sections of paraffin-embedded nude mice tumors, previously fixed with Bouin-Hollande solution, deparaffinized and rehydrated, and primary human pituitary adenoma cultures were incubated (4 C, 18 h) with antibodies against p16^INK4a^ (1:50, Santa Cruz Technologies), pRb (1:50, Cell Signalling Technology) or c-myc (1:50, Santa Cruz Technologies). After washing with PBS, samples were incubated (45 min, room temperature) with secondary antibody Alexa Flour 647. Nuclei were stained by DAPI. MtT/S clones were fixed in 4% PFA and nuclei were stained with DAPI. All samples were mounted with Mowiol mounting medium and observed with a LSM 710 AxioObserver confocal microscope (Carl-Zeiss). For tumor sections, six independent pictures for each condition were taken. Images were acquired with ZEN 2011 software (Carl-Zeiss) and analyzed in ImageJ.

### Statistics and correlation

Statistics were performed by analysis of variance (ANOVA) in combination with the Scheffé's test or Student t test. Data are shown as mean ± SEM. Correlations between variables were evaluated by Pearson's correlation. Statistical significance was accepted at p<0.05.

## SUPPLEMENTARY FIGURES


