# Supplementary material for: Autocrine IL-6 mediates pituitary tumor senescence
Source: Oncotarget. 2016 Nov 24;8(3):4690–702. doi: 10.18632/oncotarget.13577 (PMC5354864; doi:10.18632/oncotarget.13577)
Supplement: Supplementary file 1 [file oncotarget-08-4690-s001.pdf]

Autocrine IL-6 mediates pituitary tumor senescence

SUPPLEMENTARY FIGURES

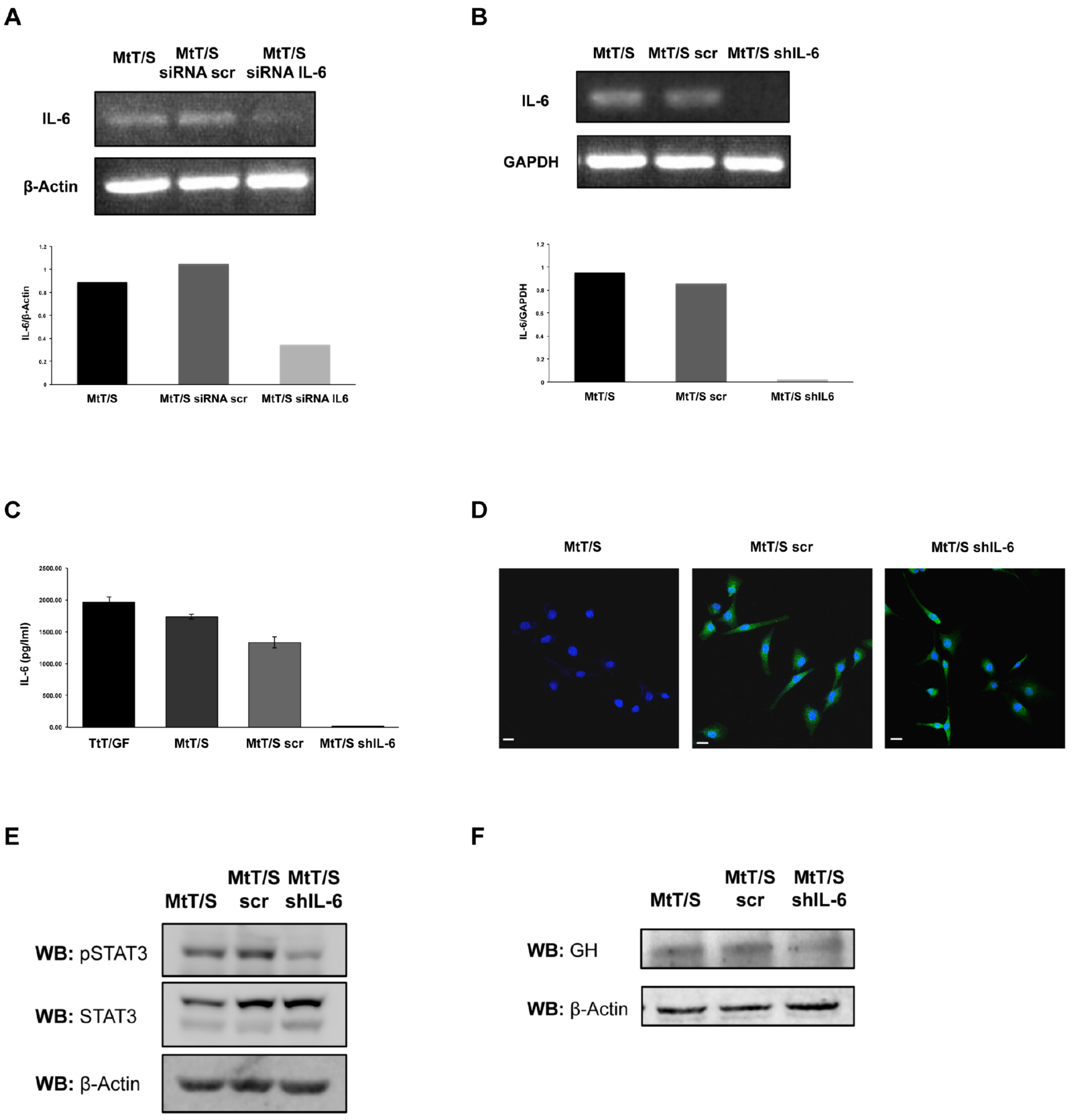

Supplementary Figure S1: Verification of IL-6 silencing in cell lines. A-B. RNA extracted from untreated MtT/S cells or MtT/S transfected with either siRNA directed toward IL-6 or unspecific siRNA (A), or MtT/S, MtT/S scr and MtT/S shIL-6 (B), was analyzed by RT-PCR and the intensity of the bands corresponding to each product was then quantified using Image J software. The value of the intensity of the band corresponding to IL-6 was normalized to its corresponding β-Actin or GAPDH band value. **C.** IL-6 secretion (300,000 cells/well) by TtT/GF, MtT/S, MtT/S scr and MtT/S shIL-6 cells under basal conditions was measured by ELISA. Results expressed as mean ± SEM of triplicates from one experiment. **D.** eGFP detection by Confocal microscopy. 4′,6-diamidino-2-phenylindole (DAPI) was used for staining cell nuclei (blue). Images show eGFP and DAPI merged.Scale bar: 20µm. **E.** For pSTAT3 and STAT3 protein levels, extracts from MtT/S, MtT/S scr and MtT/S shIL-6 cells were subjected to WB with the indicated antibodies. β-Actin was used as loading control. One MtT/S scr (#13) or MtT/S shIL-6 clones (#36), from three clones with similar results are shown. **F.** For Growth hormone (GH) protein levels, extracts from MtT/S, MtT/S scr and MtT/S shIL-6 cells were subjected to WB with the indicated antibodies. β-Actin was used as loading control. One MtT/S scr (#13) or MtT/S shIL-6 clones (#36), from three clones with similar results are shown.

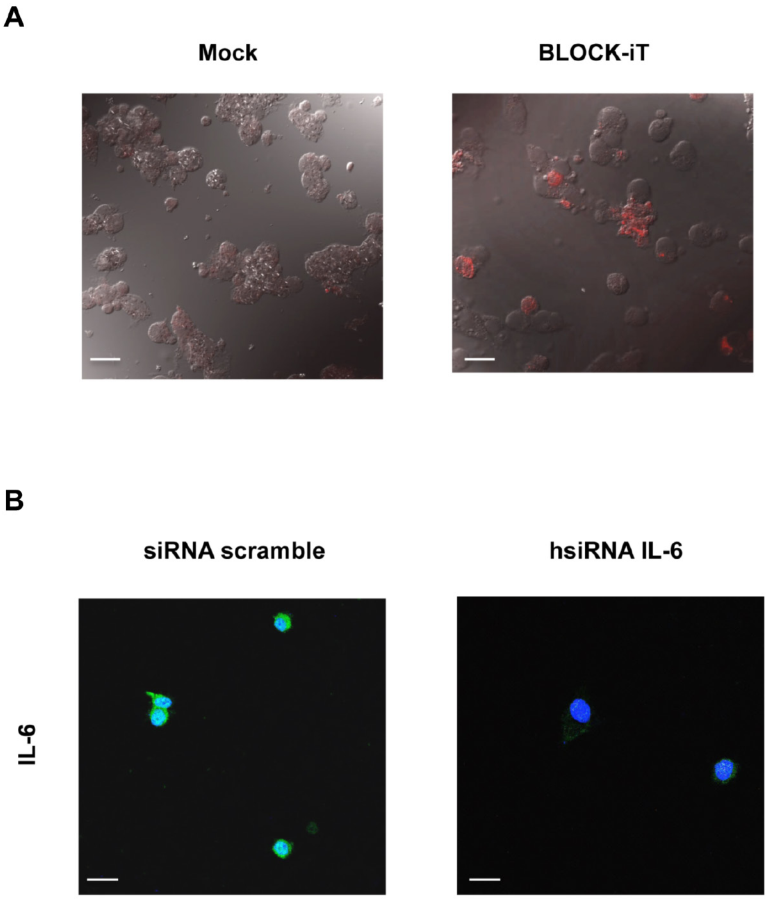

Supplementary Figure S2: Verification of IL-6 silencing in pituitary primary cell cultures. A-B. Confocal microscopy photograph of one representative primary culture (out of a total of 34 with similar results) of a human pituitary tumor sample (GH-secreting) that was either mock electroporated (left) or electroporated with a control fluorescent coupled oligonucleotide (BLOCK-iT) (right) (A), orelectroporated with hsiRNA scramble as control (left) or with hsiRNA targeting IL-6 (right), subjected to immunofluorescence with a specific antibody against IL-6 (green) (B).4′,6-diamidino-2-phenylindole (DAPI) was used for staining cell nuclei (blue). Images show green signal and DAPI merged (B).Scale bar: 20μm.
